# *Lawsonia intracellularis* in the feces of wild rodents and stray cats captured around equine farms

**DOI:** 10.1186/s12917-017-1155-8

**Published:** 2017-08-11

**Authors:** Jeong-Min Hwang, Myung-Ji Seo, Jung-Yong Yeh

**Affiliations:** 1 Veterinary Research Center, Green Cross Veterinary Products Co., Ltd., Kugal-dong 227-5, Giheung-gu, Yongin-si, Gyeonggi-do 17066 Republic of Korea; 2 Division of Bioengineering, Incheon National University, Academy-ro 119, Incheon, 22012 Republic of Korea; 3 Department of Bioengineering and Nano-Bioengineering, Graduate School of Incheon National University, Academy-ro 119, Incheon, 22012 Republic of Korea; 4 Department of Life Sciences, College of Life Sciences and Bioengineering, Incheon National University, Academy-ro 119, Yeonsu-gu, Incheon, 22012 Republic of Korea; 5 Previous address: Emerging & Exotic Diseases Research Laboratory, Foreign Animal Diseases Division, Animal, Plant, and Fisheries Quarantine and Inspection Agency, Anyang-ro 175, Manan-gu, Anyang-si, Gyeonggi-do 14089 Republic of Korea

**Keywords:** *Lawsonia Intracellularis*, Infection, Prevalence, Wildlife, Exposure, Equine proliferative enteropathy

## Abstract

**Background:**

Proliferative enteropathy is a global enteric disease of particular importance in pigs. The causative bacterium, *Lawsonia intracellularis*, has a wide range of susceptible host species. Recently, *L. intracellularis* has been recognized as an etiologic agent of an emerging enteric disease in foals called equine proliferative enteropathy (EPE). The presence of *L. intracellularis* in nonruminant wildlife has raised questions regarding the role of these species in EPE transmission.

**Results:**

This study investigated exposure to *L. intracellularis* in wild rodents and feral cats from eight farms with confirmed EPE. Serum (42) and fecal (40) samples from resident foals and fecal samples (131), intestinal mucosa tissues (14), and mesenteric lymph nodes (14) from wild and feral animals were collected for the evaluation of the farm status and the molecular detection of *L. intracellularis* following the diagnosis of EPE in index cases. Fresh feces from wild rodents and feral cats were collected from the ground while walking the premises or after trapping the animals using live traps. A total of 3 brown rats, 7 house mice, 1 striped field mouse, 2 grey red-backed voles, and 3 feral cats showed evidence of prior exposure to *L. intracellularis*.

**Conclusions:**

Our data add to increasing evidence demonstrating the potential for *L. intracellularis* transmission and infection in wild rodents and feral cats and provide possible evidence of interspecies transmission. The exposure of wild rodents and feral cats provides potential evidence for the spillover of *L. intracellularis* to wildlife species and raises the question of spillback to horses. Additionally, these animals may represent an indicator of environmental exposure or may be actively involved in the transmission of *L. intracellularis* to foals by acting as potential reservoir/amplifier hosts. This study is the first to demonstrate the magnitude of *L. intracellularis* shedding in the feces of wild rodents and feral cats and to indicate the significant infection risk that wild rodents and feral cats pose to naïve horses in South Korea.

**Electronic supplementary material:**

The online version of this article (doi:10.1186/s12917-017-1155-8) contains supplementary material, which is available to authorized users.

## Background

*Lawsonia intracellularis* is the etiologic agent of porcine proliferative enteropathy (PPE). PPE is considered a disease of particular importance in the pig because it usually affects growing pigs and has a large impact on performance [1]. Proliferative enteropathy, which is also known to be due to enteric disease induced by *L. intracellularis* infection, was first described in 1931 in pigs [2]. Since that time, the microbe itself or its DNA has been identified in numerous warm-blooded species [3–7] and in chickens [8] but never in humans [9, 10]. *L. intracellularis* has also been recognized as an etiologic agent in an enteric disease in foals called equine proliferative enteropathy (EPE) [11, 12], and an increasing number of clinical cases have been reported in horses [11, 13].

At present, the transmission of *L. intracellularis* is generally thought to occur through the ingestion of feed or water contaminated with *L. intracellularis*-infected feces from free-living or domestic animals [4]. Regarding transmission within a herd or between herds, wild animals can contribute to endemic infections in livestock and to the introduction, reintroduction and maintenance of pathogens [14]. For endemic diseases such as EPE, the source of introduction to foals may not be known, and the extent of wildlife contribution to local spread is largely unexplored.

Previous studies have shown that a variety of wild and domestic animals, including house mice, brown rats, striped field mice, yellow-necked mice, and common voles in pig farms in the Czech Republic [6], wild rats in Australian pig farms [15], and dogs, jackrabbits, opossums, skunks and coyotes in horse farms located in California and Kentucky [16], can shed *L. intracellularis* on farms with diagnosed PPE and EPE cases. However, no previous assessment of evidence or magnitude demonstrate of *L. intracellularis* shedding in the feces of wild rodents and feral cats has been conducted in South Korea. Thus, from the perspective of a vector/reservoir for *L. intracellularis*, the goal of the present study was to expand recent epidemiological findings to wild and feral animals from farms with EPE in an attempt to determine their role in the spread of *L. intracellularis* in South Korea.

## Methods

### Farm selection

Criteria for farm selection in this study were followed according to the previous study performed in Unites States of America [17]. Farms were chosen based on voluntary involvement following the diagnosis of EPE in foals on the basis of age, clinical signs, hypoalbuminemia, a thickened small intestinal wall, and the detection of *L. intracellularis* by PCR or serology [11, 12]. Farmers were explained the study purposes and procedures and upon agreeing to participate, they provided a written consent prior to study procedures and sample collection from their animals or captured animals. Within 7–10 days of diagnosing EPE in index cases, each farm was visited to begin the collection of study samples. A total of 8 breeding farms in South Korea were enrolled over a 26-month period (Table 1). Blood was collected from 3 to 8 resident foals from each horse farm by direct venipuncture, and fecal samples were collected from every five foals and tested for *L. intracellularis* DNA by real-time PCR. Blood for the serological analysis was collected from 42 resident foals. Fecal samples were collected from 40 resident foals. After collection, the samples were kept on ice and processed within 24 h.

**Table 1:** Serology and fecal shedding of resident foals in the farms investigated in this study tested for *Lawsonia intracellularis* (LI)

Farm ID	Location	Date diagnosing EPE	Foal serum samples (seropositive/total)	Fecal samples from foals (PCR positive/total)	Mean number of LI shed per gram of fecal samples	Main clinical findings in weanling foals at the occurrence of EPE
A	Gyeonggi	January 2011	2/5 (40%)	1/5 (20%)	1.2 × 10^5^	Anorexia, fever (rectal temperature > 38.5 °C), weight loss, watery diarrhea
B	Gangwon	December 2012	1/3 (33%)	1/5 (20%)	1.0 × 10^3^	Lethargy, anorexia, weight loss, and watery diarrhea (fecal staining of distal limb)
C	Chungnam	September 2011	3/4 (43%)	2/5 (40%)	1.4 × 10^4^	Lethargy and diarrhea varying from cow pie to watery (fecal staining of distal limb)
D	Chungnam	February 2013	1/6 (17%)	1/5 (20%)	1.2 × 10^4^	Mild lethargy, anorexia, fever, severe weight loss, and watery diarrhea
E	Jeonbuk	November 2012	1/4 (25%)	1/5 (20%)	1.3 × 10^5^	Peripheral edema (ventrum, sheath, and distal limbs), weight loss, and diarrhea
F	Jeonnam	December 2012	6/7 (86%)	3/5 (60%)	1.7 × 10^6^	Lethargy, fever (rectal temperature > 38.5 °C), severe weight loss, and diarrhea varying from cow pie to watery
G	Gyeongnam	January 2011	4/5 (80%)	2/5 (40%)	1.2 × 10^5^	Fever (rectal temperature > 38.5 °C), peripheral edema (ventrum and distal limbs), severe weight loss, watery diarrhea
H	Jeju	February 2013	6/8 (75%)	4/5 (80%)	1.7 × 10^7^	Lethargy, anorexia, fever (rectal temperature > 38.5 °C), severe weight loss, and watery diarrhea

All farms involved in this study had known occurrences of equine proliferative enteropathy (EPE)

### Animals

Fresh feces from free-living animals were collected after trapping the animals using live traps. The traps were laid in trap-lines within the farm’s boundaries, inside and outside of the horse stable, and in the farm’s surroundings in a 0.5-km radius. The surrounding habitats included agricultural fields (seasonal vegetation, rice paddies, and vegetable patches), groves, roadside ditches, forests, riversides, and low mountains. The interval between two traps was more than at least approximately 1 m. The live traps were baited with pieces of bacon, carrot, apple, and acorn. The traps were set for durations of one to three nights and checked every day. The bait was refreshed every second day.

After determining the species, the trapped animals, including grey red-backed voles (*Myodes rufocanus*), Eurasian flying squirrels (*Pteromys volans*), and Eurasian red squirrels (*Sciurus vulgaris coreae*), were released unharmed, and the feces dropped in the cage during the confinement period were collected. Trapped animals determined to be pests regulated under the authority of the Korea Ministry of Health and Welfare (Infectious Disease Prevention and Management Act) were euthanized. A small number of animals (4 brown rats (*Rattus norvegicus caraco Pallas*), 7 house mice (*Mus musculus*), 3 grey red-backed voles, and 1 striped field mouse (*Apodemus agrarius*) were killed by professional trappers to control pests on certain farms independent of the traps set for this study. When the animals were killed as part of routine rodent control, the animals were stored at −20 °C on the farm. Frozen rodents were thawed, and their intestines were removed from the body cavity. The intestinal mucosa was scraped with the blade of a sterile scalpel to obtain approximately 0.2–0.3 g of both tissue and feces. When animals were euthanized because they were classified as pests, the intestinal mucosa (ileum, cecum, and, colon) was scraped as mentioned above. The mesenteric lymph nodes were taken for DNA isolation and real-time PCR assays if the intestinal tissues or mesenteric lymph nodes were sufficient for sampling. Samples were collected under sterile conditions from each animal and from each part of the intestine (or mesenteric lymph node). The scraped mucosa or mesenteric lymph node was combined with 2 mL of sucrose-phosphate-glutamate solution with 5% fetal bovine serum and homogenized in a blender for 2 min. All cats trapped during the study period were anesthetized and neutered or spayed in accordance with local regulations (Trap-Neuter-Return policy), and released unharmed. Fecal samples were collected at the same time. To prevent potential cross-contamination between fecal and intestinal tissue samples, separate disposable gloves were worn for each collected sample. When possible, the samples were immediately sent to the laboratory and examined. Otherwise, the samples were kept at −20 °C at the farm until proper testing could be undertaken in the laboratory.

All animal handling, trapping, euthanasia and blood collection procedures were conducted in compliance with the regulations of the “Animal Care and Use Manual” of the Animal, Plant, and Fisheries Quarantine and Inspection Agency (No. 75/2011) and the “Animal Protection Law” of the Ministry of Agriculture, Food and Rural Affairs (No. 10310/2010).

### Serology

Sera from the resident foals were used to measure *L. intracellularis*-specific antibodies in the immunoperoxidase monolayer assay (IPMA) [18] in order to obtain information on EPE status of the farms involved in this study. The cultivation of *L. intracellularis* and serology using the IPMA technique were performed as described previously [18–20]. The pathogenic isolate PHE/KK421 (Korean Collection for Type Cultures 10686BP, Daejeon, South Korea) was used to infect murine fibroblast-like McCoy cells (American Type Culture Collection CRL 1696, VA, USA). Briefly, a *L. intracellularis* culture plate was incubated with sera diluted 1:60 in phosphate-buffered saline (PBS) for 30 min at 37 °C and washed 5 times with PBS (pH 7.2). Peroxidase-labeled goat anti-horse IgG was diluted 1:500 (KPL, MD, USA) in 2% bovine serum albumin and 0.08% Tween 80 in PBS and then added at a concentration of 50 μL/well. The plate was incubated for 45 min at 37 °C. The plate was washed again, and chromogen (3-amino-9-ethyl-carbazole, Dako Corporation, CA, USA) solution was added to each well. Then, the plate was incubated at room temperature for 20 min. The plate was washed with distilled water 3 times, allowed to dry, and examined using a BX50 microscope (Olympus, Tokyo, Japan). Positive samples had red-labeled bacteria in both the cytoplasm of the infected McCoy cells and the extracellular space [21–24].

### DNA extraction

Feces or intestinal tissues collected in the field that arrived at the laboratory were kept refrigerated at 4 °C prior to processing for nucleic acid purification within 48 h of collection. First, 2 mL of PBS was added to 0.2 g of feces or intestinal tissue homogenate in a conical tube. In case of fecal samples, the samples were vortexed for 10 s and centrifuged at 16,000×*g* for 1 min to remove fecal debris. To minimize contamination, all pipetting steps were performed in a laminar flow cabinet. Next, 200 μL of PBS and feces (or tissue homogenate) was processed for DNA purification using a BioRobot M48 workstation apparatus (Qiagen, GmBH, Hilden, Germany) with a MagAttract DNA Mini M48 Kit (Qiagen) according to the manufacturer’s recommendations. One negative extraction sample of other bacterial cells (*E. coli*) and 1 positive extraction sample of *L. intracellularis* were included in each experiment to check for any contamination in the DNA extraction process. The DNA concentrations were measured on the NanoDrop ND-1000 v.3.1 Spectrophotometer (NanoDrop Technologies, Inc., USA) according to the manufacturer’s instructions. The concentrations of pure chromosomal DNA were used to calculate the genome equivalents (GEs) used in the standard curves. Nucleic acids were eluted in 50 μL of buffer and stored at −70 °C.

### DNA amplification

All purified DNA samples were assayed for the presence of the aspartate ammonia lyase (aspA) gene of *L. intracellularis* by real-time PCR. All purified DNA samples from the feces or tissue homogenates were assayed in triplicate for the presence of the *L. intracellularis* aspA gene by real-time PCR as described previously (Additional file 1: Appendix 1) [16]. This real-time TaqMan PCR assay used is based on the detection of a specific 104-base pair product of the aspA gene of *L. intracellularis* (GenBank accession no. AM180252).

Precautions were taken to minimize contamination during the precipitation, preamplification, and amplification steps, including performing all pipetting steps in a laminar flow cabinet and including positive (DNA from cell-grown *L. intracellularis*) and negative (*L. intracellularis*-free DNA from fecal samples) DNA controls. Furthermore, swabs were taken from centrifuges, laminar flow cabinets, and countertops and assayed for the *L. intracellularis* aspA gene by real-time PCR to assess potential contamination. A real-time PCR assay that targeted a universal sequence of the bacterial 16S rRNA gene was used as a quality control (i.e., efficiency of DNA purification and amplification) and as an indicator of fecal inhibition as described previously [25, 26].

### Bacterial quantification

The amount of *L. intracellularis* in the feces was determined. For the absolute quantification of *L. intracellularis*, a standard curve was generated from 10% horse feces that was negative by real-time PCR and spiked with a 10-fold dilution of the reference strain *L. intracellularis* (strain PHE/KK421) as described previously [27]. Standard curves were made by spiking 0.9 mL of 10% *L. intracellularis*–free equine feces with a 0.1 mL suspension of the reference *L. intracellularis* PHE/KK421 strain (derived from cell culture in McCoy cells (mouse fibroblast cells)) in 10-fold dilutions prior to DNA extraction. The bacterial numbers were assessed by direct counting under a microscope after indirect immunoperoxidase staining using the *L. intracellularis*-specific antibody O6 [28]. Three μL of extracted DNA was used as a template in the real-time PCR assays. Each subsequent real-time PCR experiment included the same reference concentrations of pure DNA in triplicate, which facilitated the adjustment of the standard curves to each new real-time PCR run [27]. The final quantitation for each sample was expressed as the average of the triplicate results.

### Repeatability

The biological repeatability was determined by taking double samples of feces from 16 *L. intracellularis*-positive samples as described previously [27]. The fecal or intestinal tissue samples were diluted to 10% in PBS. DNA was also extracted using a BioRobot M48 workstation apparatus (Qiagen) with a MagAttract DNA Mini M48 Kit and subsequently analyzed by real-time PCR as parallel samples. The technical repeatability was determined by measuring the concentration of *L. intracellularis* in one DNA extract from spiked feces in parallel real-time PCR samples.

### Statistical analysis

Statistical significance was set at the 5% level, and two-sided *p*-values were calculated for the analysis of the correlation between the data from the farms and the detection rates of *L. intracellularis* DNA in fecal samples from the captured wild rodents and feral cats. The statistical analysis was performed via the paired *t*-test, and a *p*-value <0.05 was accepted as significant. All data were analyzed using the GraphPad PRISM software (version 6.07 for Windows; GraphPad Software Inc.).

## Results

All farms enrolled in this study experienced EPE between September and February from 2011 to 2013. The proportion of tested foals with antibodies against *L. intracellularis* in each farm ranged from 17 to 86%, and the proportion of foals with fecal shedding in each farm ranged from 20 to 80% (Table 1). For this study, sampling was conducted for 2–3 months on each farm. Feces were collected for the bacterial shedding analysis from a total of 131 free-living animals from 8 farms with diagnosed EPE cases. The total number of captured animals in each farm ranged from 9 to 26. The highest proportion of positive wild animals was identified on farm F (25%), whereas relatively low proportions of positive animals were found on farms C, E, and H (less than 12%) (Table 2). No positive wild animals were identified on farms B or D. All animals with evidence of exposure to *L. intracellularis* in this study were clinically normal when the feces were sampled. In most cases, the feces were formed, firm, and typical of the particular species. The feces were soft (diarrheic) in one case from a feral cat captured on farm A.

**Table 2:** Wild rodents and feral cats tested for *Lawsonia intracellularis* (LI) from eight horse farms with known occurrences of equine proliferative enteropathy

Farm ID	Total no. of samples (positive/total)	Sampling period (start-end)	Family	Species	Common name	No. of samples (positive/total)
A	2/15 (13.3%)	January 2011	Muridae	*Rattus norvegicus caraco Pallas*	Brown Rat	2/5
		-		*Apodemus agrarius*	Striped Field Mouse	0/5
		February 2011	Cricetidae	*Myodes rufocanus*	Grey Red-backed Vole	0/3
			Sciuridae	*Pteromys volans*	Eurasian Flying Squirrel	0/1
			Felidae	*Felis catus*	Feral Cat	0/1
B	0/8 (0%)	December 2012	Muridae	*Rattus norvegicus caraco Pallas*	Brown Rat	0/3
		-		*Mus musculus*	House Mouse	0/2
		February 2013		*Pteromys volans*	Eurasian Flying Squirrel	0/1
			Felidae	*Felis catus*	Feral Cat	0/2
C	2/17 (11.8%)	September 2011	Muridae	*Rattus norvegicus caraco Pallas*	Brown Rat	0/1
		-		*Mus musculus*	House Mouse	1/6
		October 2011		*Apodemus agrarius*	Striped Field Mouse	0/4
			Cricetidae	*Myodes rufocanus*	Grey Red-backed Vole	0/4
			Sciuridae	*Sciurus vulgaris coreae*	Eurasian Red Squirrel	0/1
			Felidae	*Felis catus*	Feral Cat	1/1
D	0/10 (0%)	February 2013	Muridae	*Mus musculus*	House Mouse	0/7
		-	Cricetidae	*Myodes rufocanus*	Grey Red-backed Vole	0/2
		April 2013	Felidae	*Felis catus*	Feral Cat	0/1
E	1/13 (7.7%)	November 2012	Muridae	*Mus musculus*	House Mouse	1/2
		-		*Apodemus agrarius*	Striped Field Mouse	0/6
		January 2013	Cricetidae	*Myodes rufocanus*	Grey Red-backed Vole	0/4
			Sciuridae	*Sciurus vulgaris coreae*	Eurasian Red Squirrel	0/1
F	4/16 (25.0%)	December 2012	Muridae	*Rattus norvegicus caraco Pallas*	Brown Rat	1/2
		-		*Mus musculus*	House Mouse	2/7
		January 2013		*Apodemus agrarius*	Striped Field Mouse	0/4
			Cricetidae	*Myodes rufocanus*	Grey Red-backed Vole	0/1
			Felidae	*Felis catus*	Feral Cat	1/2
G	4/26 (15.4%)	January 2011	Muridae	*Rattus norvegicus caraco Pallas*	Brown Rat	0/4
		-		*Mus musculus*	House Mouse	1/7
		February 2011		*Apodemus agrarius*	Striped Field Mouse	1/6
			Cricetidae	*Myodes rufocanus*	Grey Red-backed Vole	1/5
			Sciuridae	*Sciurus vulgaris coreae*	Eurasian Red Squirrel	0/1
			Felidae	*Felis catus*	Feral Cat	1/3
H	3/26 (11.5%)	February 2013	Muridae	*Rattus norvegicus caraco Pallas*	Brown Rat	0/6
		-		*Mus musculus*	House Mouse	2/8
		April 2013		*Apodemus agrarius*	Striped Field Mouse	0/6
			Cricetidae	*Myodes rufocanus*	Grey Red-backed Vole	1/3
			Felidae	*Felis catus*	Feral Cat	0/3

Significant associations (*p* < 0.05) were found between the proportions of positive wild animals among the total animals captured from each horse farm enrolled in this study and the serological results for each farm. The proportion of positive wild animals was somewhat correlated with the positive rate in fecal samples from foals from each farm (*p* = 0.051). However, the correlation between the mean number of *L. intracellularis* shed per gram of fecal sample and the detection rate of *L. intracellularis* DNA in the fecal samples from the captured wild animals in each horse farm was not significant.

The prevalence of PCR-positive animals varied substantially between farms. *L. intracellularis* DNA was detected by PCR in the feces of 3 out of the 21 examined brown rats, 7 out of the 39 examined house mice, 1 of the 31 examined striped field mice, 2 of the 22 examined grey red-backed voles, and 3 of the 13 examined feral cats from a total of 6 horse farms (Table 3). The *L. intracellularis* DNA-positive proportion of each animal species except for the feral cats was significantly associated with the serological results and the fecal shedding rates of the resident foals (*p* < 0.005).

**Table 3:** Total numbers of wild rodents and feral cats caught at eight horse farms and the positive rates of *Lawsonia intracellularis* assessed using real-time PCR

				Correlation (*p* value)		
Family	Species	Common name	No. of samples (positive/total)	Positive percentage in captured animals vs. serology of resident foals	Positive percentage in captured animals vs. fecal shedding rate of resident foals	Positive percentage in captured animals vs. mean number of LI shed per gram of fecal samples
Muridae	*Rattus norvegicus caraco Pallas*	Brown Rat	3/21 (14%)	0.0052	0.0364	*ns* ^a^
	*Mus musculus*	House Mouse	7/39 (18%)	0.0134	0.0423	*ns*
	*Apodemus agrarius*	Striped Field Mouse	1/31 (3%)	0.0009	0.0034	*ns*
Cricetidae	*Myodes rufocanus*	Grey Red-backed Vole	2/22 (9%)	0.0008	0.0009	*ns*
Sciuridae	*Pteromys volans*	Eurasian Flying Squirrel	0/2 (0%)	-	-	-
	*Sciurus vulgaris coreae*	Eurasian Red Squirrel	0/3 (0%)	-	-	-
Felidae	*Felis catus*	Feral Cat	3/13 (23%)	*ns*	*ns*	*ns*
Total			16/131 (12%)			

^a^ *ns*, no statistically significant association between two evidences

Statistical significance was set at the 5% level, and two-sided *p*-values were calculated for the analysis of the correlation between data from the farms and the detection rates of *L. intracellularis* DNA in fecal samples from captured wild rodents and feral cats

A large number of *L. intracellularis* was shed (more than 1 × 10^7^/g of feces) by a rat from farm F and a mouse from farm G. A small proportion of wild rodents from farms F and H shed 10^5^–10^7^ *L. intracellularis* per gram of feces (Additional file 1: Appendix 2). However, the majority of the wild rodents trapped on the 6 horse farms shed less than 10^5^ *L. intracellularis*/g of feces, and *L. intracellularis* could not be detected in more than 90.0% of the wild rodents trapped on farms B, C, D, and E. All feral cats that tested positive for *L. intracellularis* shedding shed 10^5^–10^7^ *L. intracellularis* per gram of feces.

Gastrointestinal tissue samples from 11 animals that tested PCR-positive for *L. intracellularis* in the feces were investigated (Table 4). The adenomatous lesions typical of *L. intracellularis* infection were not observed in any of the necropsied animals investigated in this study. Intestinal mucosal tissues and mesenteric lymph nodes were not available for testing in another five animals that tested PCR-positive for *L. intracellularis* because the animals were released unharmed after the fecal samples were collected.

**Table 4:** The detection of *Lawsonia intracellularis* DNA by real-time PCR in the intestinal mucosal tissues and mesenteric lymph nodes of 10 wild mammals with confirmed *L. intracellularis*-positive test results in fecal samples that were available for additional tests

	Ileum			Cecum			Colon			MLN^b^			No. of examined animals		
Animal species	*n.*	Pos^a^	%	*n.*	Pos	%	*n.*	Pos	%	*n.*	Pos	%	*n.*	Pos	%
Brown Rat (*Rattus norvegicus caraco Pallas*)	3	0	0.0	3	1	33.3	3	0	0.0	3	1	33.3	3	2	66.7
House Mouse (*Mus musculus*)	7	2	28.6	7	1	14.3	7	2	28.6	7	2	28.6	7	3	42.9
Striped Field Mouse (*Apodemus agrarius*)	1	1	100	1	0	0.0	1	0	0.0	1	0	0.0	1	1	100
Total	11	3	27.3	11	2	18.2	11	2	18.2	11	3	27.3	11	6	54.5

^a^Positive; ^b^In some animals, corresponding mesenteric lymphatic nodes (MLN) were examined

Two of the 6 horse farms in which brown rats were captured and tested had brown rats with evidence of exposure to *L. intracellularis*. Four of the 7 horse farms in which house mice were captured and tested had house mice with evidence of exposure to *L. intracellularis*. In contrast, only 2 of the 6 horse farms in which grey red-backed voles were captured and tested had grey red-backed voles with evidence of exposure to *L. intracellularis*. Although striped field mice were captured on 6 horse farms, only 1 farm had striped field mice with evidence of exposure to *L. intracellularis*. Likewise, 3 farms had feral cats with evidence of exposure to *L. intracellularis*, although only 7 feral cats were captured.

Based on fecal shedding and the detection of *L. intracellularis* DNA by real-time PCR in the intestinal mucosal tissue and mesenteric lymph node samples, a total of 3 brown rats from 2 farms, 7 house mice from 5 farms, 1 striped field mouse from 1 farm, 2 grey red-backed voles from 2 farms, and 3 feral cats from 3 farms had evidence of prior exposure to *L. intracellularis*. On farms F, G, and H, 25%, 15%, and 12% of the captured wild mammals had evidence of exposure to *L. intracellularis*, respectively. The largest number of PCR-positive *L. intracellularis* fecal samples was observed in the house mice (7), followed by brown rats (3), feral cats (3), grey red-backed voles (2) and striped field mice (1).

## Discussion

Our results correlate with previous findings demonstrating similar exposure rates to *L. intracellularis* in weanling foals from farms with EPE in a study on horse farms located in California and Kentucky, with a serological prevalence for each farm that ranged from 11 to 100% [16]. Our finding also indicates that the exposure rates to *L. intracellularis* in foals from farms with EPE were moderate to high at the time point when the first clinical EPE case were diagnosed on each farm.

Based on individual identification or the detection of only 1 positive animal for a specific species, an exact prevalence of 23% was determined for feral cats and 3% for striped field mice. Among the wild animals captured in this study, the highest detection rate of *L. intracellularis* DNA was found in feral cats. The relatively high prevalence determined for feral cats may be influenced by the small sample size; thus, additional samples are needed to assign a more accurate prevalence. However, this study showed a low detection rate of *L. intracellularis* DNA in the total number of striped field mice (3%) compared with a previous report on the prevalence in wild rodents from pig farms with PPE (16%) [6]. None of the fecal samples from Eurasian flying squirrels or Eurasian red squirrels had detectable *L. intracellularis*. The lack of molecular pathogen detection in these species may be related to the small sample size, a potential intermittent mode of pathogen shedding, or the inability of *L. intracellularis* to infect these species as previously described [16].

Collectively, three rodent species and 1 feral cat species were positive for *L. intracellularis* at farms A, C, E, F, G, and H, suggesting that the bacterium had been introduced into the surrounding environments of these particular farms. The presence of *L. intracellularis* in wild rodents and feral cats living in close proximity to the farm led us to assume that this pathogen might be shed into the natural environment and spread as previously described [6]. The transmission of the bacterium may occur not only between wild rodents or feral cats themselves but also to other free-living animals, such as wild boars, deer and carnivores.

From the perspective of herd prevalence in wild animals, the proportion of positive feces from the wild animals captured in this study ranged from 0 to 25% for each farm. Differences in habitat, the number of trapping events between farms, and the total numbers of each captured animal species between the farms might be at the source of this discrepancy. Additionally, the results of this study highlight the variety of animal species that are potentially involved in the shedding of *L. intracellularis* on farms with documented EPE cases in South Korea. Based on our results, a number of free-living rodents and feral cats can be considered host or reservoir species of *L. intracellularis*. Furthermore, we identified a new *L. intracellularis-*infected host: the grey red-backed vole. This species may be an important vector/reservoir of *L. intracellularis* in the Far East region, including South Korea, because this species ranges across northern Eurasia, including the Korean Peninsula, and is frequently found in South Korea.

The parallel finding of the causative agent in the intestinal mucosal membrane and the corresponding mesenteric lymph nodes in one of the brown rats and two of the house mice was suggestive of the earlier findings in domestic pigs [29]. Our observations seem to suggest that *L. intracellularis* infection among wild rodents and feral cats around horse farms can spread through the oro-fecal route similar to domestic pigs on farms with intensive breeding [30].

The importance of EPE transmission by vectors is unknown. Following experimental inoculation, histological lesions develop in laboratory mice, rats and hamsters but not in sparrows or chickens [6, 7, 15, 31–34]. Natural infection has been described in rats and mice, but the importance of these vectors for transmission within a herd or transmission between herds under natural conditions is uncertain. However, rats, mice, and cats have been considered important reservoirs of *L. intracellularis* on pig or horse farms, with the prevalence of PCR-positive animals varying substantially between farms from 4 to 83% [6, 7, 15, 17, 31].

Evidence of exposure to *L. intracellularis* in wild rodents and feral cats captured on farms with EPE was higher than previously reported, possibly due to differences in regions or sampling methods; for instance, surveys reporting prevalence rates of 1.0 and 0%, respectively, have used PCR analysis of tissue or fecal samples, respectively [17, 35].

A random sampling-based study previously performed in South Korea reported that 15.7 and 12.5% of tissue samples from wild rodents and stray cats, respectively, tested PCR-positive for *L. intracellularis* [31]. Because the detailed species investigated in this previous study were not determined, information on the prevalence of each rodent species was limited. Interestingly, the overall rates of *L. intracellularis* DNA detection in wild rodents and feral cats were not significantly different, although the research design in terms of farm selection was different between our study and the study by Truong et al. This difference might explain why the previous study by Troung et al. investigated animals captured on pig farms regardless of consideration of farm selection based on PPE outbreak by *L. intracellularis* infection, which was in contrast to our method. Additionally, Lee et al. reported an extraordinarily high nationwide prevalence of PPE-positive pig farms (100%) in South Korea [36].

Regardless of the affected species, the antemortem diagnosis of proliferative enteropathy is based on the detection of *L. intracellularis*–specific IgG by serology and the molecular identification of *L. intracellularis* DNA in feces by PCR [22, 37]. Positive serology characterizes exposure to infection rather than disease, whereas positive PCR results indicate shedding of the bacteria and active infection. However, to date, epidemiologic studies that have determined the exposure rate of free-living animals to *L. intracellularis* have been hampered by the lack of established and validated serological assays [16]. Difficulties associated with in vitro cultivation of this organism related to its ubiquitous presence and ability to cause disease in a variety of animal species also highlight the need for higher resolution diagnostics to provide a better understanding of the interspecies transmission dynamics and the realistic importance of the disease in different species [38]. Among a variety of diagnostic methods, the molecular detection of *L. intracellularis* in the feces of animals generally does not provide any conclusive evidence regarding the biological state of the organism or its origin. The direct link between wild animals and EPE still needs to be proven by either characterization of the detected bacterial isolates or experimental challenges using isolates from free-living hosts. To overcome these difficulties, good diagnostic methods and increased knowledge of epidemiology and immunity are required for the *L. intracellularis* bacterium.

A major limitation of this study was the lack of widely-accepted serological assay to evaluate *L. intracellularis*-specific antibodies in cat or rodent species. Although IPMA could be available in our laboratory in this study and actually performed for sera from some animals, the serological data for cats and rodents is not shown in this paper because the assay was not fully evaluated and still not widely accepted for sera from cats and rodents. Another limitation was the study design, which was based on voluntary enrollment instead of randomized selection. Similar limitations were reported by Pusterla et al., [16] in the United States. Likewise, no farms with EPE over the study period refused participation in this study. In addition, one of the limitations of this study was that the study lacked control farms. However, we did not try to determine the risk factors associated with the occurrence of EPE but instead focused solely on the exposure rates of *L. intracellularis* in wild and feral animals.

The mode of transmission of *L. intracellularis* to susceptible weanlings remains speculative. However, foals most likely become exposed to *L. intracellularis* after the ingestion of feed or water contaminated by *L. intracellularis*-containing feces from domestic or free-living animals. Potential reservoir hosts must be abundant on the premises, have unlimited access to feeding and drinking areas of the susceptible weanlings, and be able to maintain the agent indefinitely within their populations. From the study results, brown rats, house mice, striped field mice, grey red-backed voles, and feral cats would be considered prime reservoir host candidates in South Korea. At the very least, these wild animal species may play a role in the circulation of *L. intracellularis* in the natural habitats around EPE-affected horse farms in the Far East, including South Korea. Additional domestic or free-living animals might have played a role in the transmission of *L. intracellularis* on the horse farms enrolled in this study. Therefore, the role of clinically and subclinically infected foals in the feco-oral transmission of *L. intracellularis* requires further investigation.

## Conclusions

In summary, the infection source of *L. intracellularis* in foals remains speculative worldwide, and only limited information on epidemiological findings in wild and feral animals from farms with EPE has been reported in the United States in an attempt to determine their role in the spread of *L. intracellularis*. This study is the first report to describe the identification of five wild mammalian hosts that are potentially associated with the shedding of *L. intracellularis* on farms with a known EPE status in South Korea. Additionally, this study is the first to demonstrate the magnitude of *L. intracellularis* shedding in the feces of wild rodents and feral cats and to indicate the significant infection risk that wild rodents and feral cats pose to naïve horses in South Korea. These results also demonstrate the importance of understanding the role of wildlife species in the development of management strategies for EPE in weanling foals in South Korea. The findings emphasize the need to enforce biosecurity measures to prevent wild animals, including wild rodents and feral cats, from entering horse stables.

## Additional file

Additional file 1: Appendix 1. Oligonucleotide primers and probe used for the real-time PCR detection of Lawsonia intracellularis in the fecal samples used in this study. **Appendix 2.**The total percentages of wild and feral animals shedding Lawsonia intracellularis and the percent shedding at high (more than 1 × 10^7^), medium (10^5^–10^7^) or low (10^3^–10^5^) numbers of L. intracellularis/g of feces or with undetectable numbers in each horse farm. (DOC 100 kb)

## Abbreviations

EPE: Equine proliferative enteropathy
IPMA: Immunoperoxidase monolayer assay
PBS: Phosphate-buffered saline; aspA: aspartate ammonia lyase
PCR: Polymerase chain reaction
PPE: Porcine proliferative enteropathy
