# Supplementary material for: Lawsonia intracellularis in the feces of wild rodents and stray cats captured around equine farms
Source: BMC Vet Res. 2017 Aug 11;13:233. doi: 10.1186/s12917-017-1155-8 (PMC5553581; doi:10.1186/s12917-017-1155-8)
Supplement: Additional file 1: Appendix 1. — Oligonucleotide primers and probe used for the real-time PCR detection of Lawsonia intracellularis in the fecal samples used in this study. Appendix 2.The total percentages of wild and feral animals shedding Lawsonia intracellularis and the percent shedding at high (more than 1 × 10^7^), medium (105–107) or low (103–105) numbers of L. intracellularis/g of feces or with undetectable numbers in each horse farm. (DOC 100 kb) [file 12917_2017_1155_MOESM1_ESM.doc]

Appendix 1. Oligonucleotide primers and probe used for the real-time PCR detection of *Lawsonia intracellularis* in the fecal samples used in this study

| Target gene | Assay | Primer/probe | Oligonucleotide sequences | Reference |
| --- | --- | --- | --- | --- |
| *L. intracellularis*  aspartate ammonia lyase gene | Real-time TaqMan PCR  with preamplification | Forward | AATTTGTTGTGGATTGTATTCAAGGA |  |
|  |  | Reverse | CTTTCTCATGTCCCATAAGCTCAA |  |
|  |  | Probe | 6FAM-CAGGGACAAGTACAAATATGAATGCTAATGAAGCAA |  |

Appendix 2. The total percentages of wild and feral animals shedding *Lawsonia intracellularis* and the percent shedding at high (more than 1 x 10^7^), medium (10^5^–10^7^) or low (10^3^–10^5^) numbers of *L. intracellularis*/g of feces or with undetectable numbers in each horse farm.

| Farm ID | No. of  rats tested (positive/total) | No. of  mice^†^ tested (positive/total) | No. of  squirrels^‡^ tested (positive/total) | No. of feral  cats tested (positive/total) | Total %  PCR positive animals | Percentage of rodents shedding *L. intracellularis* at a range of concentrations | | | |
| --- | --- | --- | --- | --- | --- | --- | --- | --- | --- |
|  |  |  |  |  |  | >1 x 10^7^/g feces (%) | 10^5^–10^7^/g feces (%) | 10^3^–10^5^/g feces (%) | Undetected (%) |
| A | 2/5 |  |  |  | 40.0 | 0 | 0 | 40.0 | 60.0 |
|  |  | 0/8 |  |  | 0 | 0 | 0 | 0 | 100 |
|  |  |  | 0/1 |  | 0 | 0 | 0 | 0 | 100 |
|  |  |  |  | 0/1 | 0 | 0 | 0 | 0 | 100 |
| B | 0/3 |  |  |  | 0 | 0 | 0 | 0 | 100 |
|  |  | 0/2 |  |  | 0 | 0 | 0 | 0 | 100 |
|  |  |  | 0/1 |  | 0 | 0 | 0 | 0 | 100 |
|  |  |  |  | 0/2 | 0 | 0 | 0 | 0 | 100 |
| C | 0/1 |  |  |  | 0 | 0 | 0 | 0 | 100 |
|  |  | 1/14 |  |  | 7.1 | 0 | 0 | 7.1 | 92.9 |
|  |  |  | 0/1 |  | 0 | 0 | 0 | 0 | 100 |
|  |  |  |  | 1/1 | 100 | 0 | 100 | 0 | 0 |
| D |  | 0/9 |  |  | 0 | 0 | 0 | 0 | 100 |
|  |  |  |  | 0/1 | 0 | 0 | 0 | 0 | 100 |
| E |  | 1/12 |  |  | 8.3 | 0 | 0 | 8.3 | 91.7 |
|  |  |  | 0/1 |  | 0 | 0 | 0 | 0 | 100 |
| F | 1/2 |  |  |  | 50.0 | 50.0 | 0 | 0 | 50.0 |
|  |  | 2/12 |  |  | 16.7 | 0 | 8.3 | 8.3 | 83.3 |
|  |  |  |  | 1/2 | 50.0 | 0 | 50.0 | 0 | 50.0 |
| G | 0/4 |  |  |  | 0 | 0 | 0 | 0 | 100 |
|  |  | 3/18 |  |  | 16.7 | 5.6 | 0 | 11.1 | 83.3 |
|  |  |  | 0/1 |  | 0 | 0 | 0 | 0 | 100 |
|  |  |  |  | 1/3 | 33.3 | 0 | 33.0 | 0 | 66.7 |
| H | 0/6 |  |  |  | 0 | 0 | 0 | 0 | 100 |
|  |  | 2/17 |  |  | 11.8 | 0 | 5.9 | 5.9 | 88.2 |
|  |  |  |  | 0/3 | 0 | 0 | 0 | 0 | 100 |
| †House mice (*Mus musculus*), striped field mice (*Apodemus agrarius*), and grey red-backed voles (*Myodes rufocanus*) were examined; ‡Eurasian flying squirrels (*Pteromys volans*) and Eurasian red squirrels (*Sciurus vulgaris coreae*) were examined. | | | | | | | | | |
